# A viral infection prediction model for patients with r/r B-cell malignancies after CAR-T therapy: a retrospective analysis

**DOI:** 10.3389/fonc.2025.1549809

**Published:** 2025-03-21

**Authors:** Shujing Guo, Jile Liu, Bing Wang, Xiaomei Zhang, Yifan Zhao, Jianmei Xu, Xinping Cao, Mohan Zhao, Xia Xiao, Mingfeng Zhao

**Affiliations:** ^1^ First Center Clinical College, Tianjin Medical University, Tianjin, China; ^2^ Department of Hematology, Tianjin First Central Hospital, Tianjin, China; ^3^ School of Medicine, Nankai University, Tianjin, China; ^4^ Department of Hematology, Hebei University Affiliated Hospital, Baoding, Hebei, China

**Keywords:** refractory/relapsed B cell malignances, chimeric antigen receptor T cells, predictive models, viral infection, granulocyte colony-stimulating factor

## Abstract

**Background:**

Chimeric antigen receptor T cell (CAR-T) therapy for relapsed/refractory (r/r) B cell acute lymphoblastic leukemia (B-ALL) and B cell non-Hodgkin lymphoma (B-NHL) patients has shown promising effects, but side effects such as viral infections have been observed.

**Methods:**

A total of 45 patients with r/r B-ALL and r/r B-NHL were included in this retrospective study. Patient demographics were recorded, with the primary endpoint being viral infection within 3 months post CAR-T treatment. Univariate and multivariate logistic regression analyses and least absolute shrinkage and selection operator (LASSO) regression analysis were used to analyze independent factors. The patients were divided into a training cohort of 28 and a validation cohort of 17 to construct a prediction model based on determined independent factors. The model’s discrimination and calibration were assessed using the receiver operating characteristic curve (ROC), calibration plot, and decision curve analysis (DCA curve).

**Results:**

The univariate and multivariate logistic regression analyses of the 43 patients showed that low baseline lymphocyte ratio was an independent risk factor and using granulocyte colony-stimulating factor (G-CSF) early was a protective factor for viral infection after CAR-T therapy in patients with B-ALL and B-NHL. Based on that, the area under the ROC curve (AUC) of the training cohort and validation cohort was 0.935 (95% CI 0.837-1.000) and 0.869 (95%CI 0.696-1.000), respectively, showing excellent predictive value.

**Conclusions:**

We established a nomogram to predict the factors’ influence on viral infection after CAR-T therapy and found that the ratio of baseline lymphocytes and using G-CSF early or lately were able to predict viral infection after CAR-T therapy in r/r B-ALL and B-NHL.

## 1 Introduction

Chimeric antigen receptor T cell (CAR-T) therapy, as an attractive immunotherapy in recent years, has shown high efficacy in B cell malignancies and especially in relapsed/refractory (r/r) B cell acute lymphoblastic leukemia (B-ALL) (1–3). There are mature commercial products targeting CD19 to treat B-ALL and B cell non-Hodgkin lymphoma (B-NHL) (4).

Although CD19 CAR-Ts are a promising treatment tactic, as they have shown magnificent efficacy, the side effects of this product should not be ignored. Apart from acute toxicities including cytokine release syndrome (CRS) and immune effector cell–associated neurotoxicity syndrome (ICANS) (5, 6), infection risk should not be neglected, which leads to the “on target, off-tumor” depletion of normal CD19-expressing B cells and malignancy prior to treatment and lymphodepletion chemotherapy (3, 7, 8).

Viral infections occur after CD19 CAR-T treatment frequently, with respiratory viral infection being the most common, followed by viral reactivation, such as hepatitis viruses (including HBV and HCV) and HSV. Other double-stranded viruses such as adenovirus and BK polyomavirus also occur in patients from time to time (7, 9–11). The infection risk after CAR-T therapy is influenced by the whole phase of immune reconstruction. There is a high incidence of viral infections 1 month after a CAR-T infusion, which correlates with neutropenia and anti-inflammatory treatments (7, 12). Subsequent destruction of humoral immunity and persistent B cell aplasia and hypogammaglobulinemia, which continue to affect the patient’s antiviral immunity, lead the patient to be more susceptible to viruses. These effects can last for several months or even more (13–15).

Viral infection is a common complication associated with CD19 CAR-T therapy, significantly impacting the long-term survival of patients. Therefore, empowering clinical practitioners to predict and avert the occurrence of viral infections in patients following CAR-T therapy is conducive to enhancing the therapeutic effects in patients. Currently, the knowledge regarding the risk factors of viral infection following CD19 CAR-T therapy for B-lymphocyte malignancies is relatively insufficient. Based on previous research on infections after CAR-T therapy, we collected relevant risk factors of patients with B cell malignancies (B-ALL and NHL) who received CD19 CAR-T therapy at Tianjin First Central Hospital (ChiCTR-ONC-16008911) to identify and evaluate the risk factors associated with viral infection after CD19 CAR-T therapy, and construct prediction models and perform clinical decision curve analyses to assess the accuracy and clinical value of the models.

## 2 Methods

### 2.1 Patients and study design

**Figure 1:**
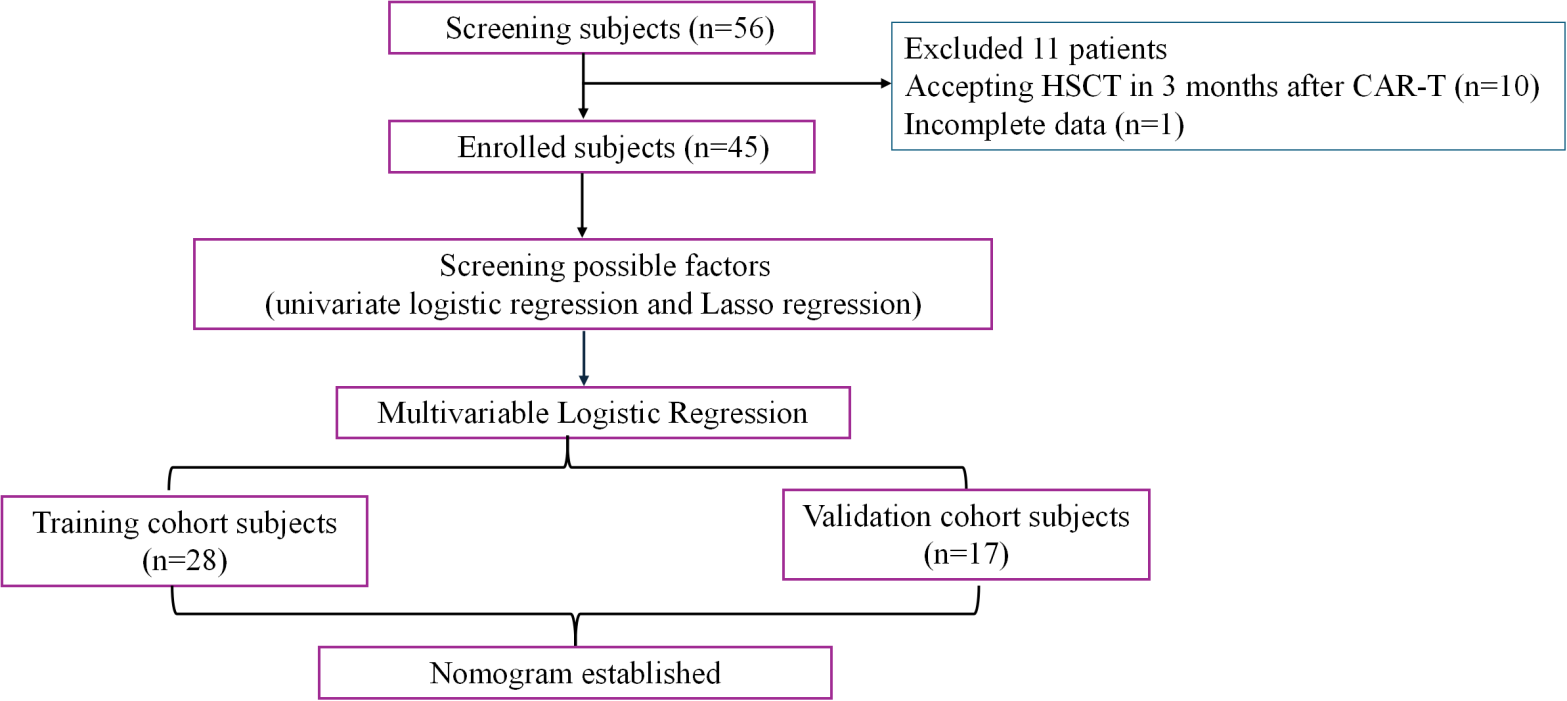
The workflow of this study.

A total of 56 patients with r/r B-ALL and B-NHL who received CD19 CAR-T therapy at Tianjin First Central Hospital from January 2022 to December 2023 were retrospectively collected. Among them, there were 34 patients with r/r B-ALL and 22 with r/r B-NHL. The inclusion criteria are as follows: (1) patients diagnosed with r/r B-ALL or NHL according to the WHO 2016 diagnostic criteria (16); (2) autologous or allogeneic T cells were selected to prepare the CAR-T therapy; (3) medical records within 6 months of CAR-T treatment were complete and accessible. In total, 10 patients (nine with B-ALL and one with B-NHL) were excluded due to receiving hematopoietic stem cell transplantation (HSCT) within 3 months after CD19 CAR-T therapy while one patient was excluded because of incomplete data. Thus, a total of 45 patients were included in this analysis. Among them, 27 patients were used as the training cohort, and another 18 patients were the validation cohort. The process of this study is presented in **Figure 1**.

### 2.2 Data collection

**Figure 2:**
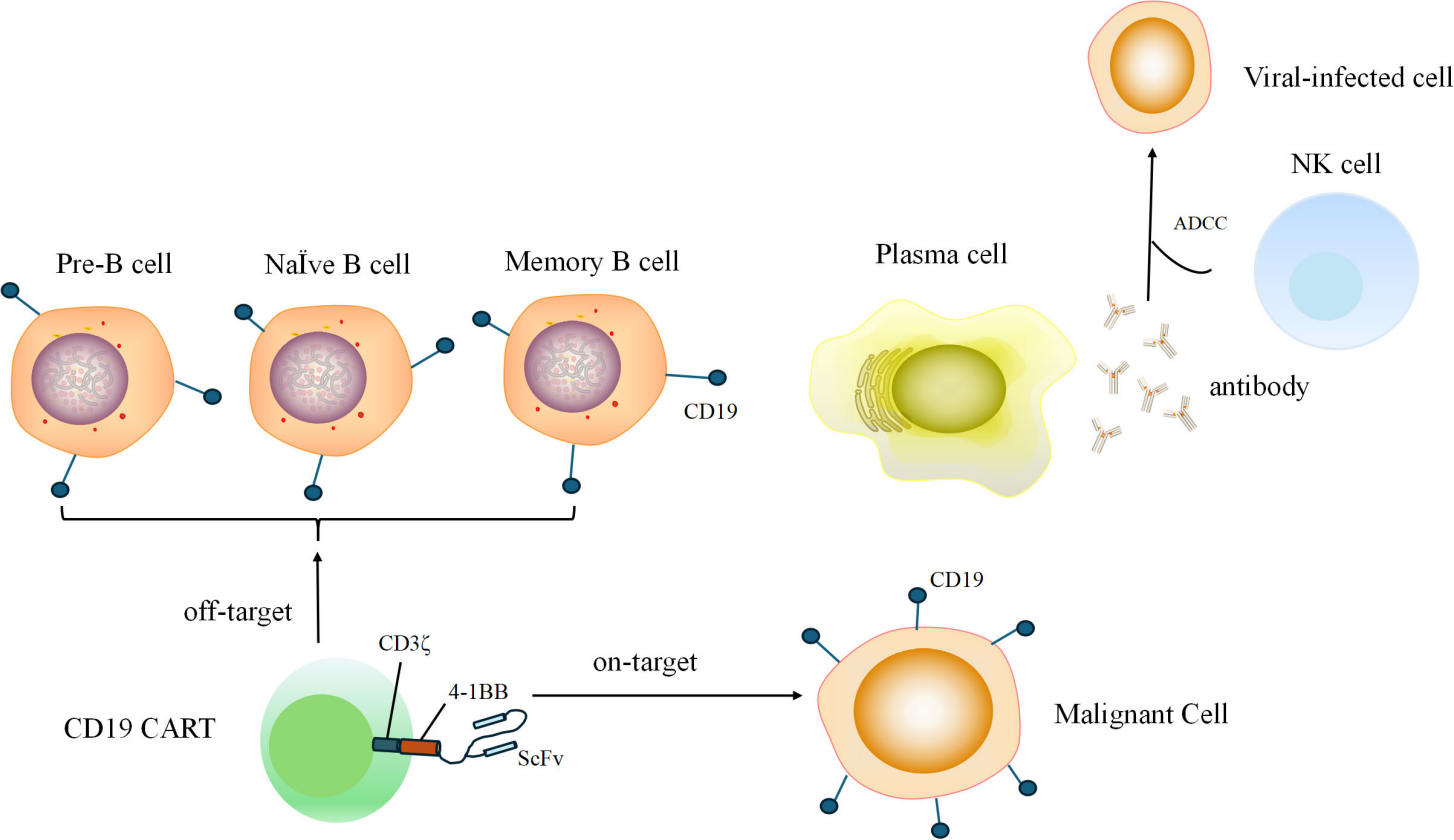
During B cell developmental and differentiation processes, pre-B cells, naive B cells, and memory B cells express CD19. Memory B cells differentiate into plasma cells, which secrete antibodies to kill viral-infected cells through the ADCC. CD19 CAR-T that attack normal CD19-expressing B cells are called “off-target”.

We selected possible risk factors associated with the incidence of viral infection after CD19 CAR-T treatment (9–15). One of the most important reasons for susceptibility to viruses is the “on target, off-tumor” depletion of normal CD19-expressing B cells. The mechanism of this process is shown in **Figure 2**. Thus, we collected data including baseline hematological parameters, disease burden, information on the CAR-T infusion, and the treatment strategy after CAR-T infusion. The endpoint was viral infection after a CD19 CAR-T infusion within 3 months or not (including emerging viral infection and virus reactivation). We use viral nucleic acid amplification testing to evaluate viral infection. Before CAR-T infusion, we tested the patients for hepatitis B, hepatitis C, syphilis, Acquired Immune Deficiency Syndrome, Epstein–Barr virus, cytomegalovirus, and varicella-zoster virus infections. If the hepatitis B virus DNA level is above 500 IU/mL, entecavir is taken continuously for at least 4 weeks until the virus turns negative. Thereafter, antiviral drugs for hepatitis B are taken regularly for prevention. After CAR-T cell infusions, we regularly perform viral nucleic acid amplification testing on patients. When patients experience clinical symptoms such as fever and cough, viral nucleic acid amplification testing is performed to determine whether the patient has a viral infection. The subtypes of the infected virus are shown in **Supplementary Table 1**. After testing positive for a virus, patients receive active antiviral treatment depending on the type of virus. For patients infected with COVID-19, antiviral treatment should be carried out as early as possible to actively correct hypoxemia. According to the actual clinical situation, hormone therapy and an infusion of human immunoglobulin therapy are administered. In the process of grouping continuous variables, we categorized the treatment lines into two groups at four lines. The median number of treatment lines is four lines, so we categorized treatment lines into two groups: <4 lines and ≥4 lines. For the proportion of lymphocytes in white blood cells, we evaluated it 5 days before chemotherapy pretreatment and used 20% as the cut-off because, in a blood routine test, the normal proportion of lymphocytes is set at 20% to 40%. When the proportion of lymphocytes is lower than this normal proportion, it is associated with the occurrence of infections. For peak cytokine levels after CAR-T therapy, we utilized 100pg/ml as the reference point and classified the patients into three groups for analysis: <100pg/ml, 100 pg/ml to 1,000 pg/ml, and ≥1,000 pg/ml (17, 18). Early use of granulocyte colony-stimulating factor (G-CSF) was defined as using G-CSF within 14 days after a CAR-T infusion.

### 2.3 Development and assessment of the nomogram

Univariate logistic regression (entry criterion: P<0.05) and least absolute shrinkage and selection operator (LASSO) regression were used to assess the association between clinical characteristics and the incidence of viral infection after CD19 CAR-T therapy. Independent predictors were assessed using multivariate logistic regression and then used to develop a nomogram for predicting viral infections after CAR-T therapy within 3 months. The receiver operating characteristic (ROC) curve, the area under the ROC curve (AUC), and the concordance index (C-index) were used to assess the ability of the nomogram to predict viral infections. Calibration curves were used to assess the agreement between the actual observations and the nomogram predictions. Decision curve analysis (DCA) was conducted to assess the sensitivity and specificity of the dataset and thus the clinical utility. The ROC curve, calibration curve, and DCA curve were applied in the validation cohort and training cohort.

### 2.4 CAR-T therapy

CD19 CAR-T therapy is an investigational product that was manufactured in our center. The CD19 CAR vector includes a scFv FMC63-based targeting domain, CD8-derived hinge, transmembrane domain, human 4-1BB costimulatory domain, and CD3ζ signaling domains. The whole construction is loaded into the pCDH-MSCV-MCS-EF1-T2A-Puro vector. Compared with CD19 CAR-Ts with CD28 as the co-stimulatory domain, CD19 CAR-Ts containing 4-1BB have a more persistent anti-tumor effect. The vector was then transduced into T cells with a certain amount of lentivirus. T cells were isolated from the blood of patients or contributors with peripheral blood cell separators and CD3-coated magnetic beads (Miltenyi,130-097-043). CD3 and CD28-coated magnetic beads (Sigma, 11161-D) and interleukin-2 (IL-2; T&L Biotechnology Co., Ltd) were added to stimulate T cell expansion. Next, for CAR lentiviral transduction, we chose the multiplicity of infection (MOI) of 5. After 7–10 days of culturation, CAR-Ts were infused into patients. Before the infusion, 3 days of lymphodepleting chemotherapy with fludarabine (25 mg/m^2^/day) and cyclophosphamide (250 mg/m^2^/day) was administered.

### 2.5 Statistical analysis

Statistical software SPSS 26.0.0 was used for the univariate logistic regression analysis. All reported P-values are two-tailed and a P-value < 0.05 was considered significant and the variable was then integrated into multivariate logistic regression. The Kaplan–Meier curve, LASSO regression, multiple logistic regression, and the building of the prediction models were performed using R software (version 4.2.0). The following R packages were used: ggpubr, corrplot, glmnet, caret, CBCgrps, tidyverse, rms, and autoReg. Descriptive statistics are reported as frequencies for categorical variables and median ± standard deviation (SD) and interquartile ranges (IQR) for continuous variables.

## 3 Results

### 3.1 Adverse effects

**Figure 3:**
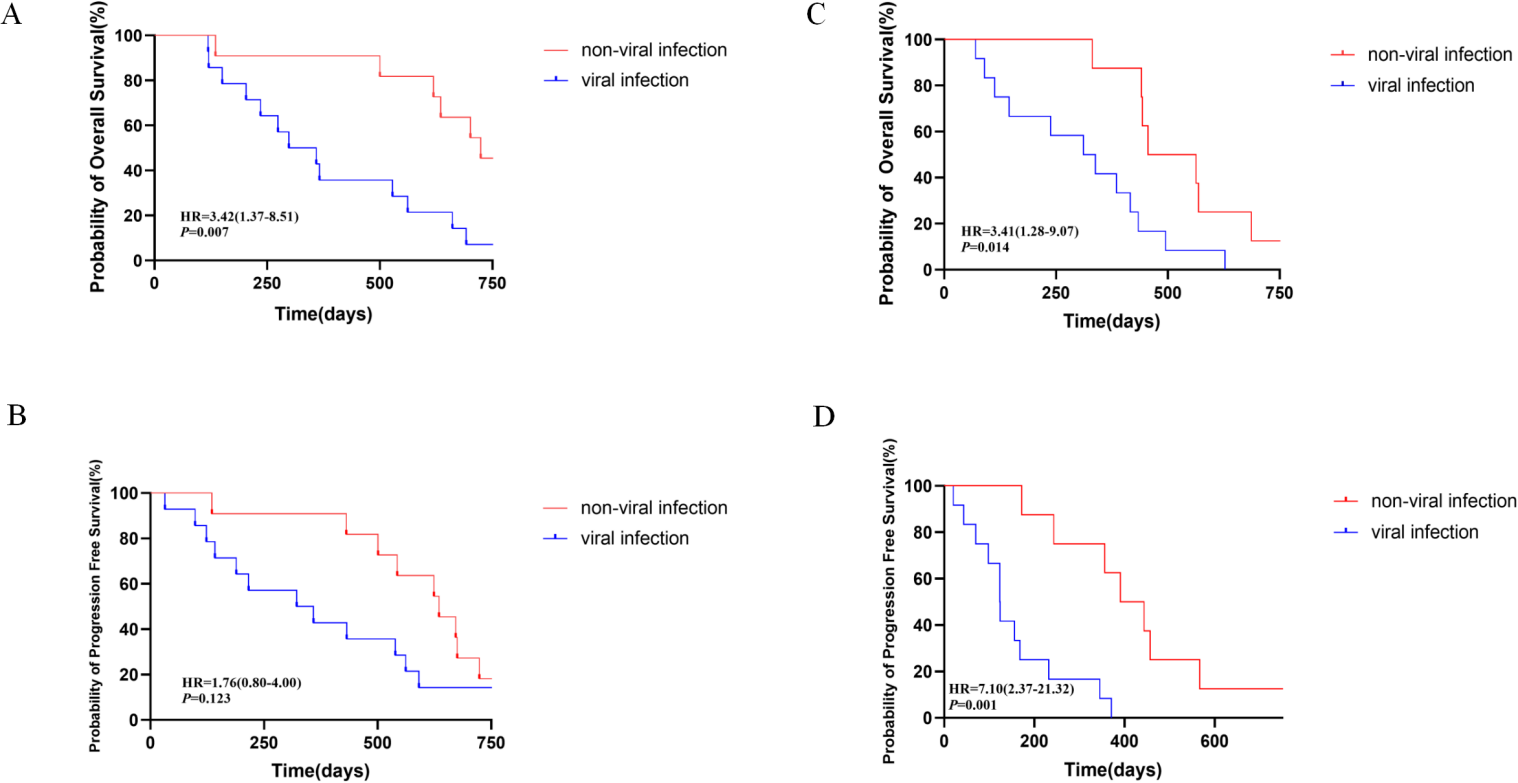
Long-term survival and outcomes of the patients enrolled in this study, with the comparisons between patients with non-viral infection and viral infections 3 months after CAR-T therapy. **(A, B)** Overall survival and progression survival of patients with ALL. **(C, D)** Overall survival and progression-free survival of patients with B-NHL.

We conducted subgroup survival analysis on patients with B-ALL and B-NHL included in the study based on whether viral infection occurred within 3 months after CAR-T therapy. The median overall survival time (OS) of the patients with ALL and B-NHL without viral infections in the 3 months after CAR-T therapy was 806 days and 563 days respectively, while that for patients with ALL and B-NHL with viral infections was 661 days and 416 days, respectively. The median progression-free survival (PFS) duration of patients with ALL with and without viral infections in the 3 months after CAR-T therapy was 562 days and 672 days, respectively, and that for patients with B-NHL with and without viral infections was 232 days and 457 days, respectively. We found that patients with B-ALL (P=0.007, HR=3.42, 1.37-8.51) and B-NHL (P=0.014, HR=3.41,1.28-9.07) who developed viral infections within 3 months after CAR-T treatment had significantly lower OS compared to patients who did not develop viral infections. In addition, B-NHL patients who developed viral infections within 3 months after receiving CAR-T cell therapy also had significantly lower PFS compared to uninfected patients (P=0.001, HR=7.10, 2.37-21.32). As shown in **Figure 3**, viral infection influences long-term survival and outcomes of patients, so building a prediction model to avoid and control viral infections after CAR-T therapy is meaningful. This outcome shows viral infection influences long-term survival and outcomes of patients, so building a prediction model to avoid and control viral infections after CAR-T therapy is meaningful. As of the date of the most recent follow-up visit, 37 patients had disease progression/relapse and 21 had died because of disease progression.

### 3.2 General characteristics

We collected data on general information, disease burden, and CAR-T therapy-related information from a total of 56 patients and excluded 11 patients, with 45 patients finally included in this study. Of these patients, 25 patients were diagnosed with r/r B-ALL and 20 with r/r B-NHL. Taking into account that there were some differences in their clinical characteristics, especially the different criteria to evaluate the disease burden, we decided to describe the general characteristics of patients with the two diseases separately.

#### 3.2.1 General characteristics of the patients with B-ALL

There were 14 patients who developed viral infections in 3 months after CAR-T therapy among the 25 patients with B-ALL. Among these eligible B-ALL cases, the median age was 45 (IQR 28-50). In total, 12(48%) patients were male and 13 (52%) were female. There were 12 (48%) ph (+) and 3 (12%) ph-like patients. Five (20%) patients had extramedullary invasion, including one case with central neurologic system (CNS) invasion. Further general characteristics data are shown in **Table 1**.

**Table 1:** General characteristics of B-ALL patients.

Variable	Total (n = 25) M (p25, p75)/n%	^1^No (n = 11) M (p25, p75)/n%	^2^Yes (n = 14) M (p25, p75)/n%	p
Age (year)	45 (28, 50)	39 (35,49)	46 (19,60)	0.833
Sex				0.529
Male	12 (48%)	4 (36%)	8 (57%)	
Female	13 (52%)	7 (64%)	6 (43%)	
Number of treatment lines				0.017
<4	9 (36%)	7 (64%)	2 (14%)	
≥4	16 (64%)	4 (36%)	12 (86%)	
Proportion of lymphocyte				<0.001
<20% 16 (64%)		2 (18%)	14 (100%)	
≥20% 9 (36%)		9 (82%)	0 (0%)	
WBC (*10^9/L) 3.49 (2.457,5.11)		2.09 (1.50,2.77)	3.14 (1.92,4.03)	0.429
HSCT before CAR-T infusion				0.099
No	10 (40%)	2 (18%)	8 (57%)	
Yes	15 (60%)	9 (82%)	6 (43%)	
Fusion gene				0.278
Ph (-)	10 (40%)	4 (36)	6 (43)	
Ph (+)	12 (48%)	7 (64)	5 (36)	
Ph-like	3 (12%)	0 (0)	3 (21)	
BM tumor burden	0% (0%, 14%)	0% (0%, 18.5%)	0.5% (0%, 12.8%)	0.904
Extramedullary invasion				0.604
No	20 (80%)	10 (91%)	10 (71%)	
^3^Yes	1 (4%)	0 (0%)	1 (7%)	
^4^CNS	4 (16%)	1 (9%)	3 (21%)	
Source of T				0.529
Autologous	12 (48%)	4 (36%)	8 (57%)	
Allogeneic	13 (52%)	7 (64%)	6 (43%)	
INF-γ				<0.001
<100	11 (44%)	1 (9%)	10 (71%)	
100-1000	8 (32%)	4 (36%)	4 (29%)	
>1000	6 (24%)	6 (55%)	0 (0%)	
^6^Hormone				0.407
No	18 (72%)	9 (82%)	9 (64%)	
Yes	7 (28%)	2 (18%)	5 (36%)	
^7^Tocilizumab				1.000
No	18 (72%)	8 (73%)	10 (71%)	
Yes	7 (28%)	3 (27%)	4 (29%)	
^9^CRP	13 (6.12, 36.06)	10.71 (6.94, 26.68)	15.52 (6.42, 44.41)	0.647
^10^G-CSF				0.009
No	13 (52%)	2 (18%)	11 (79%)	
Yes	12 (48%)	9 (82%)	3 (21%)	
Pre-existing infections				0.356
No	13 (52%)	6 (55%)	7 (50%)	
Yes	12 (48%)	5 (45%)	7 (50%)	
IL-6				0.965
<100	11 (44%)	5 (46%)	6 (43%)	
100-1000	10 (40%)	4(36%)	6 (43%)	
>1000	4 (16%)	2 (18%)	2 (14%)	

P-values were determined using the Mann–Whitney U-test and χ2 test.^1^Ending without viral infection;^2^ending with viral infection;^3^extramedullary invasion (except CNS);^4^CNS invasion; ^5^using hormones to control CRS; ^6^using tocilizumab to control CRS;^7^CRP after 14 days; ^8^early use of G-CSF.

#### 3.2.2 General characteristics of the patients with B-NHL

There were 12 patients who developed viral infections in 3 months after CAR-T therapy among the 20 patients with B-NHL. Among these eligible B-NHL cases, the median age was 48(IQR 36-62). In total, 12 (60%) patients were male and 8 (20%) were female. The median International Prognostic Index (IPI) score was 3(IQR 3-4). Four (20%) patients had CNS invasion. Further general characteristics data are shown in **Table 2**.

**Table 2:** General characteristics of B-NHL patients.

Variable	Total (n = 20)	0 (n = 8)	1 (n = 12)	p
Age	48 (36,62)	59 (48,64)	42 (36,58)	0.159
Sex				0.648
Male	12 (60%)	4 (50%)	8 (67%)	
Female	8 (40%)	4 (50%)	4 (33%)	
Number of treatment lines				0.065
<4	9 (45%)	6 (75%)	3 (25%)	
≥4	11 (55%)	2 (25%)	9 (75%)	
Proportion of lymphocyte				0.062
<20%	13 (65%)	3 (38%)	10 (83%)	
≥20%	7 (35%)	5 (62%)	2 (17%)	
WBC(*10^9g/L)	4.28 (2.88,6.11)	4.44 (3.38, 6.28)	3.70 (2.0, 6.66)	0.630
IPI	3 (3,4)	3.5 (3,5)	3 (2,4)	0.743
CNS invasion				0.619
No	16 (80%)	7 (88%)	9 (75%)	
Yes	4 (20%)	1 (12%)	3 (25%)	
HSCT before CART				0.325
No	14 (70%)	7 (88%)	7 (58%)	
Yes	6 (30%)	1 (12%)	5 (42%)	
Source of T				1.000
No	17 (85%)	7 (88%)	10 (83%)	
Yes	3 (15%)	1 (12%)	2 (17%)	
INF-γ				0.117
<100	16 (80%)	8 (100%)	8 (67%)	
100-1000	4 (20%)	0 (0%)	4 (33%)	
Hormone				0.117
No	16 (80%)	8 (100%)	8 (67%)	
Yes	4 (20%)	0 (0%)	4 (33%)	
Tocilizumab				0.117
no	16 (80%)	8 (100%)	8 (67%)	
yes	4 (20%)	0 (0%)	4 (33%)	
CRP (mg/L)	6.79 (2.13, 17.74)	3.8 (1.57, 6)	13.7 (6.1, 28.95)	0.031
G-CSF				0.019
No	12 (60%)	2 (25)	10 (83)	
Yes	8 (40%)	6 (75)	2 (17)	
Pre-existing infections				
No	10 (50%)	4 (50%)	6 (50%)	1.000
Yes	10 (50%)	4 (50%)	6 (50%)	
IL-6				1.000
<100	10 (50%)	4 (50%)	6 (50%)	
100-1000	5 (25%)	2 (25%)	3 (25%)	
>1000	5 (25%)	2 (25%)	3 (25%)	

P-values were determined using the Mann–Whitney U-test and χ2 test. The definitions of factors are the same as **Table 1**.

The baseline characteristics of the patients with B-ALL and B-NHL and the p-values are shown in two tables. We found that the factors that influenced the viral infection incidence after CD19 CAR-T therapy in both patient cohorts were similar, in particular, treatment lines, proportion of lymphocytes before CAR-T infusion, accepting HSCT before CAR-T therapy, and accepting G-CSF early after CAR-T therapy demonstrated statistical value. Furthermore, we found that factors such as BM tumor burden before CAR-T therapy, IPI score, and existing CNS invasion related to the type of diseases were associated with viral infection after CD19 CAR-T therapy without statistical significance using a preliminary Mann–Whitney U-test and χ2. We based the following analysis on the baseline characteristics of the patients with B-ALL and B-NHL by removing the factors directly related to the disease type and analyzing the patients with the two different B cell malignancies together.

### 3.3 Establishment of a prediction model

**Figure 4:**
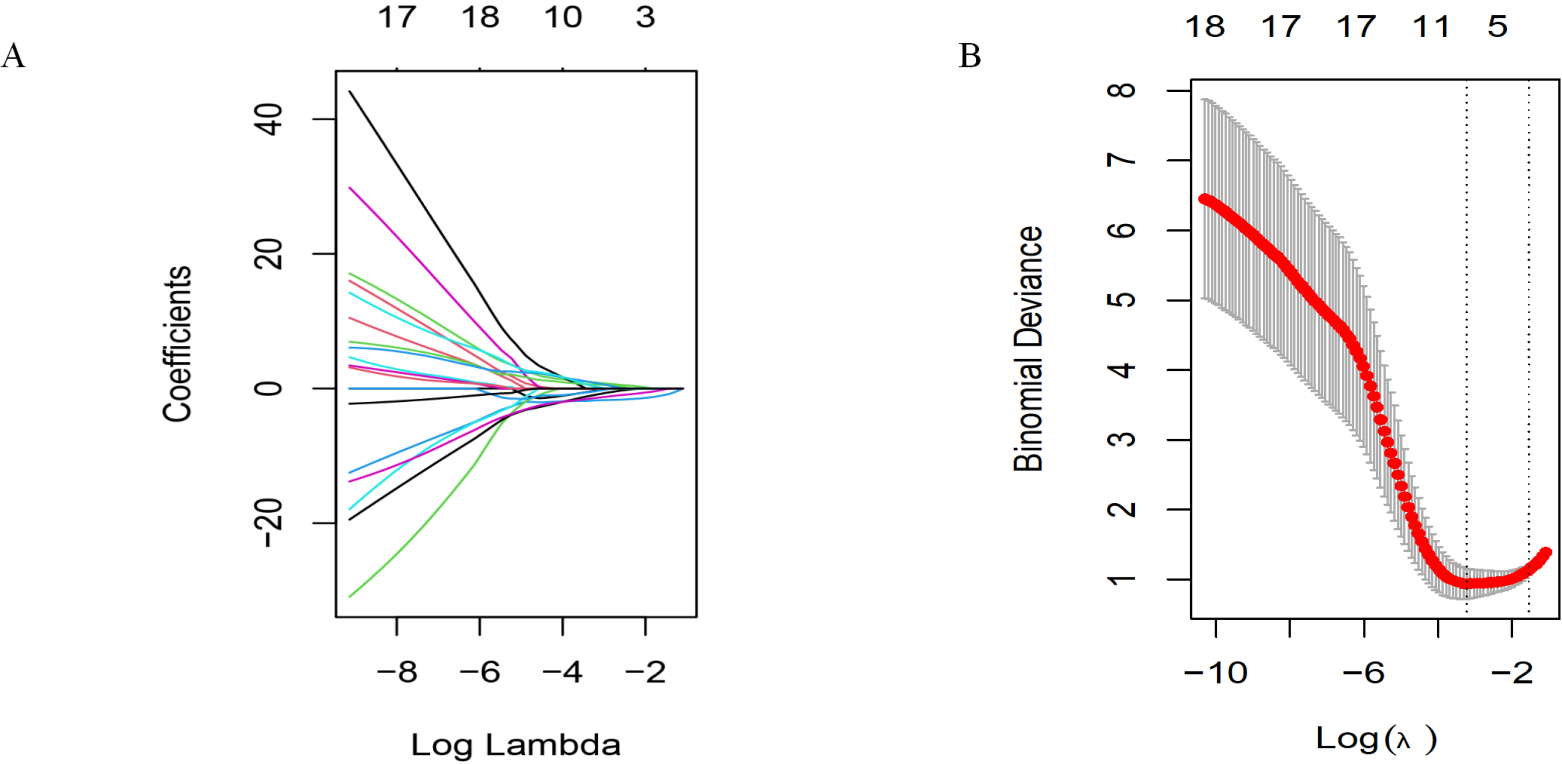
Selecting significant factors with LASSO regression. **(A)** LASSO coefficient profiles of the 19 variables. **(B)** The cross-validation results.

We first attempted LASSO regression, as shown in **Figure 4**. This is a widely used linear regression method in statistics and machine learning, particularly suitable for feature selection and high-dimensional data. To determine the optimal model parameters, we applied a 10-fold cross-validation method to iterative analysis. After multiple iterations and validations, we found that the LASSO regression model exhibited excellent performance when λ (regularization parameter) was set to 0.043436 (corresponding to a logarithmic λ value of -1.37). Incorporating 3–10 variables into the binary logistic regression analysis can achieve the best predictive performance.

Using the univariable logistic regression, we found four factors: treatment lines (P=0.002), proportion of lymphocytes (P<0.001), HSCT before CAR-T (P=0.023), and G-CSF (P<0.001).

According to the results of the LASSO regression and univariable logistic analyses, we chose treatment lines, proportion of lymphocytes, HSCT before CAR-T, and G-CSF for the multivariable logistic regression analysis.

**Figure 5:**
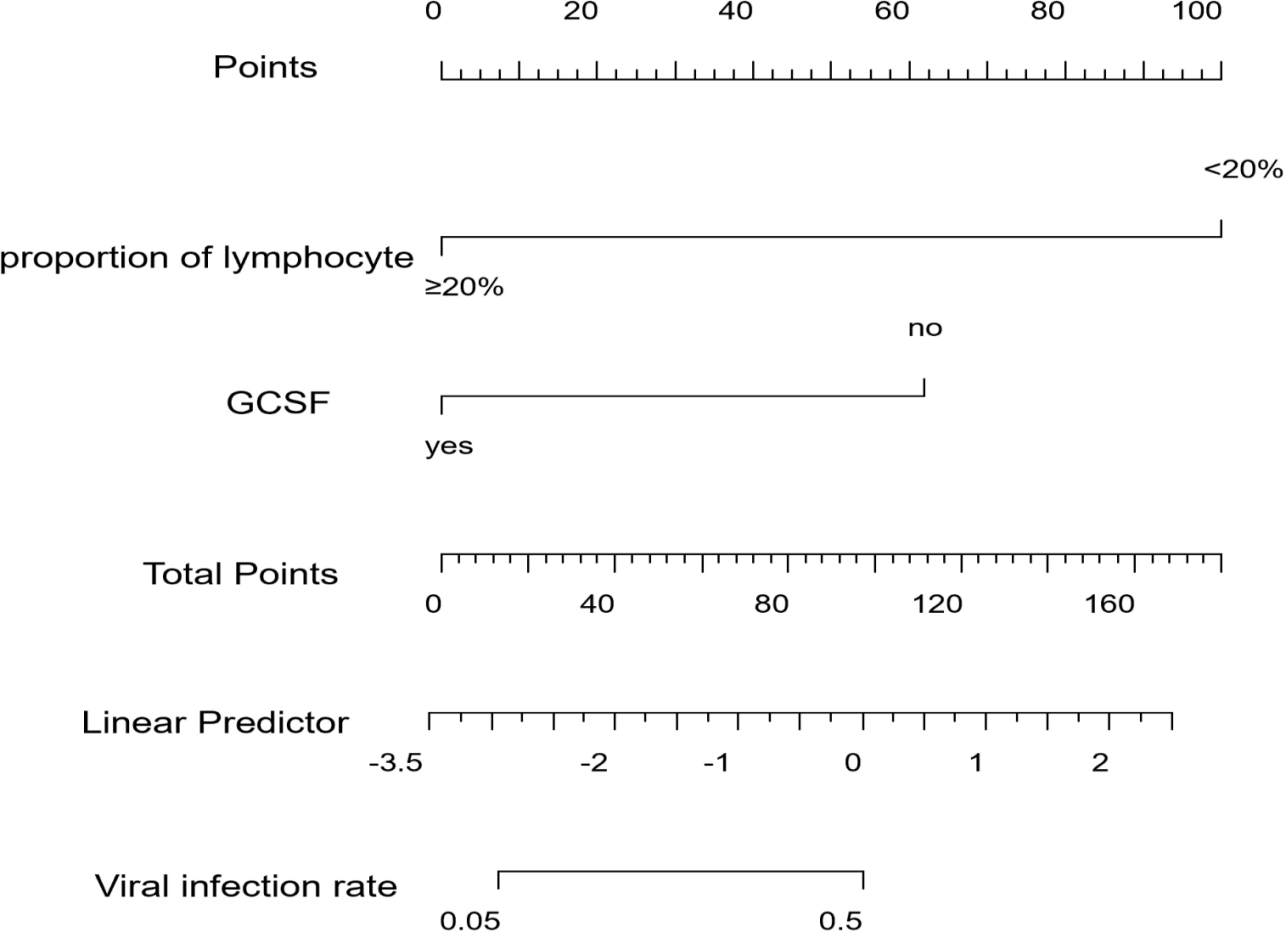
Nomogram for the prediction of viral infection after CAR-T therapy. Characteristics in the nomogram to predict viral infection after CAR-T therapy. To use the nomogram, the specific points of individual patients are located on each variable axis. Lines and dots are drawn upward to determine the points received by each variable. The sum of these points is located on the Total Points axis. A line is drawn to the “Viral infection rate” axis to determine the probability of viral infection after CAR-T therapy.

The binary logistics regression analysis results are shown in **Table 3**. We found that the proportion of lymphocytes and receiving G-CSF were independent predictors of a viral infection after CAR-T therapy. A high proportion of lymphocytes (P=0.008, HR=0.04, 95%CI 0.00-0.42) and receiving G-CSF early (P=0.032, HR=0.10, 95%CI 0.01-0.82) were negative factors for viral infection after CAR-T therapy, or can be understood as protective factors for decreasing viral infection rate after CAR-T therapy. We also created a visual nomogram to represent this predictive model (**Figure 5**). The results of LASSO regression and logistic regression can be regarded as mutual validation.

**Table 3:** The Binary logistics regression analysis results.

Variable	^1^No (n=19) n (%)	^2^Yes (n=26) n (%)	P	HR (95%CI)
^3^Treatment lines			0.337	
<4	13(68.4%)	5 (19.2%)		
≥4	6 (31.6%)	21 (80.8%)		
^4^Proportion of lymphocyte			0.008	
<20%	5 (26.3%)	2 (7.7%)		
≥20%	14 (73.7%)	10 (38.5%)		0.04 (0.00-0.42)
HSCT before CAR-T				
No	14 (73.7%)	10 (38.5%)	0.070	
Yes	5 (26.3%)	16 (61.5%)		
^5^G-CSF			0.032	
No	4 (21.1%)	21(80.8%)		
Yes	15 (78.9%)	5 (19.2%)		0.10 (0.01-0.82)

P-values and HR (95% CI) were determined using the binary logistic regression analysis. ^1^Without viral infection after CAR-T; ^2^with viral infection after CAR-T; ^3^ number of treatment lines before CAR-T therapy; ^4^the baseline proportion of lymphocytes before CAR-T of patients; ^5^recieving G-CSF early after CAR-T.

### 3.4 Validation of the prediction model

**Figure 6:**
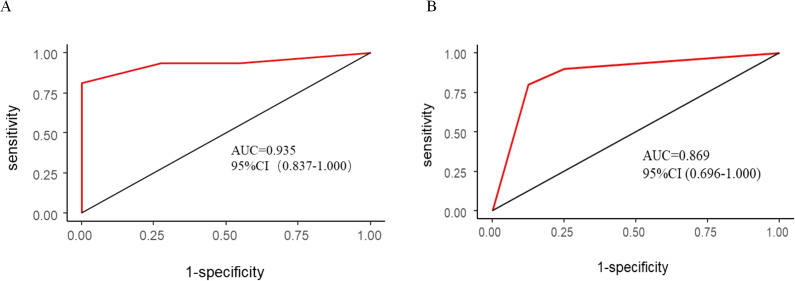
ROC curve and AUC of the nomogram. **(A)** Training cohort; **(B)** validation cohort.

In the training cohort, the AUC value was 0.935 (95% confidence interval: 0.837–1.000), indicating that the model exhibits exceptionally high discriminative power in distinguishing between positive and negative cases. In the validation cohort, the AUC value was 0.869 (95% CI: 0.696-1.000), which, although slightly lower than in the training cohort, still demonstrates robust predictive performance. An AUC value closer to 1 signifies higher prediction accuracy of the model (**Figure 6**). The ROC curve illustrates the relationship between the true positive rate (TPR) and false positive rate (FPR) for a classification model, with the AUC serving as a comprehensive measure of the model’s overall performance across different thresholds.

**Figure 7:**
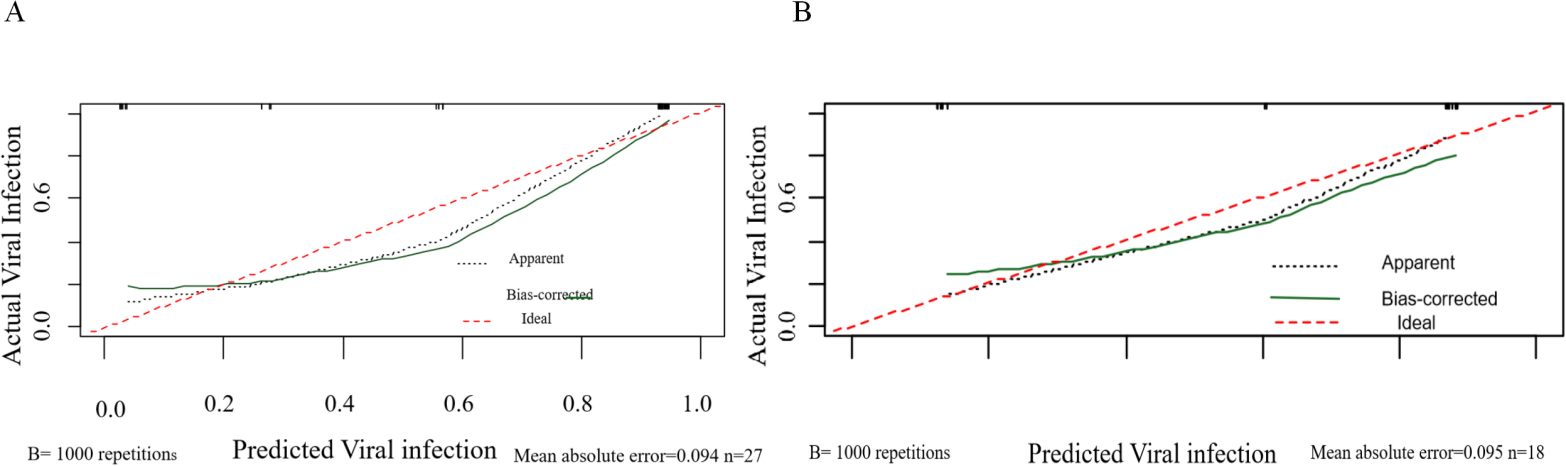
Calibration curves of the nomogram. **(A)** Calibration curves for viral infection after CAR-T therapy in the training cohort. **(B)** Calibration curves for viral infection after CAR-T therapy in the validation cohort. The red line represents the ideal reference line where the predicted probability matches the observed viral infection rate.

The calibration curve assesses the agreement between the predicted probabilities generated by the model and the actual observed probabilities. In this study, the calibration curves for both the training and validation cohorts closely approximated the ideal line (i.e., the diagonal line), indicating that the model’s predicted probabilities are reliable and there is no systematic overestimation or underestimation (**Figure 7**). This strong calibration further enhances the credibility of the model’s predictions.

**Figure 8:**
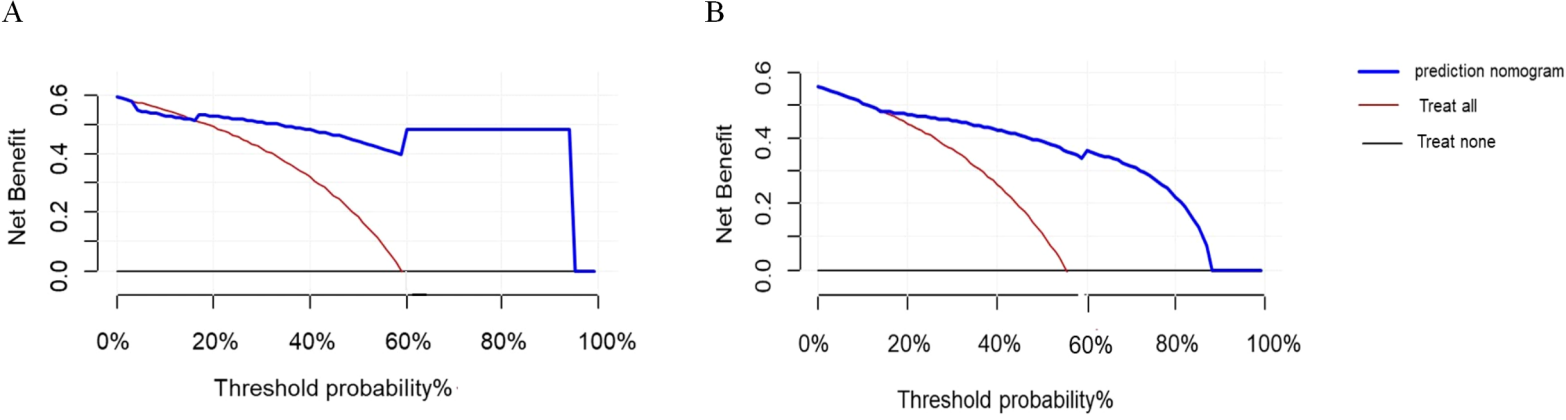
DCA curves. **(A)** DCA curve for viral infection after CAR-T therapy in the training cohort. **(B)** DCA curve for viral infection after CAR-T therapy in the validation cohort.

DCA evaluates the clinical utility of predictive models by calculating net benefits at various threshold probabilities, thereby assessing model performance under different intervention strategies. In this study, the DCA threshold range was derived from the sensitivity and specificity of the model in both the training and validation cohorts. The results demonstrated that, compared to the extreme strategies of intervening in all patients or none, the model provided higher net benefits across multiple thresholds (**Figure 8**). This suggests that the model can offer valuable guidance for clinical decision-making.

## 4 Discussion

CD19 CAR-T therapy is a promising treatment for B cell malignancies, which can bring hope for patients with r/r B-ALL or B-NHL. As CAR-T therapies are becoming routine treatments, the management and treatment of the side effects of CAR-T therapy have attracted more and more attention from physicians (19, 20). In addition to the widely known and published acute toxicity effects such as CRS and ICANs, infection after CAR-T should not be ignored. Viral infection is a risk factor affecting the survival of patients after CD19 CAR-T therapy. Patients who experience viral infection and relapse within 3 months after receiving a CD19 CAR-T infusion exhibit shorter overall survival and progression-free survival.

Viral infection and reactivation after a CD19 CAR-T infusion are challenging issues that require more experience and relevant knowledge from doctors to avoid. At present, there is less research focusing on this aspect, so clinical doctors do not have sufficient evidence to take measures to reduce the possibility of viral infection and reactivation in patients.

Thus, we attempted to build a clinical prediction model to guide physicians to prevent and reduce viral infection after CD19 CAR-T therapy. This study finally included 45 patients with B cell malignancies, including B-NHL and B-ALL. Infection after CAR-T is multifactorial. Prior treatment lines and prior HSCT are high-risk factors, which were also validated in this analysis. Using lymphodepleting chemotherapy before CAR-T therapy and immune-suppressing drugs after CAR-T therapy are regarded as risk factors for infection after CAR-T therapy. In addition to the factors mentioned above, an important factor that must be emphasized is the off-target effect of CD19 CAR-T therapy, because normal B cells, which play a significant role in humoral immunity, are attacked by CAR-Ts. The result is anti-virus immune damage which needs time to reconstruct. Finally, neutropenia is a high risk factor in many types of infections, especially after CAR-T therapy (8, 21). Before relative studies about infection after CAR-T, some risk factors were found. Hill et al. found that receiving >4 lines of treatment was associated with a higher infection rate after CAR-T therapy (22), and prior HSCT also increased infection rates after CAR-T according to an analysis by Vora et al. (23). Park et al. found that CRS was associated with a high infection rate after CAR-T therapy (24). Whether the patient had B-ALL or B-NHL, some indications associated with disease burden were not associated with our study endpoint (viral infection or reactivation after CD19 CAR-T within 3 months). According to the baseline characteristics and the previous related studies, we chose some factors in an initial selection. The univariable logistic analysis presented four factors that were significantly associated with the endpoint. Patients who received≥4 lines of treatment and prior HSCT had an increased risk of viral infection after CD19 CAR-T therapy within 3 months. Another risk factor was a low proportion of lymphocytes (<20%) in the baseline hematological profile before CAR-T therapy. A protective factor was using G-CSF early after CAR-T therapy. The P-value of all these factors was less than 0.05 in the univariable logistic regression analysis. These results are consistent with other studies. In further analysis, we included these four factors into a multivariate logistic regression, and finally found two factors, low proportion of lymphocyte and using G-CSF early, that increased and decreased the risk of viral infection after CAR-T therapy, respectively. In summary, our research provided clinical insights, such as using G-CSF to shorten the duration of neutrophil depletion, thereby avoiding the risk of viral infection in patients, especially for high-risk patients with a low proportion of lymphocytes (<20%). Neutropenia is a common side effect after CAR-T therapy and a long neutropenia duration can lead to infection. A severe viral infection caused by human herpes virus 6 (HHV-6) was documented after a long duration of neutropenia in previous clinical trials (25). Thus, on the one hand, using G-CSF early to rectify neutropenia is meaningful. On the other hand, the use of G-CSF is not recommended in the first 3 weeks after a CAR-T infusion or before CRS has resolved [43]. Some *in vivo* studies found that GM-CSF plays a key role in promoting CRS and levels of GM-CSF and G-CSF have been found to be elevated in patients with neurotoxicity (26–28). A single-center study compared patients treated with G-CSF and without G-CSF and found no increased risk of severe CRS or ICANS ^[46]^. There are also other studies that found that GM-CSF can modulate T cell activation and increase the anti-tumor function in a preclinical model (29). More research, especially prospective studies, is needed to explore the role and timing of G-CSF in CAR-T therapy.

However, this study has a few limitations. First, since the establishment of the prediction model is based on single-center research data, external validation (using CD19 CAR-T therapy datasets from different centers) can help enhance the reliability of the prediction model. Furthermore, due to the retrospective nature of the study, there may be potential biases and other confounding factors in data collection. Therefore, in future research, we will conduct prospective studies to further validate the conclusions drawn in this study. In addition, although our analysis also included factors correlated with CRS and disease burden, these indications did not significantly influence the endpoint, which may be because of the small number of patients (**Supplementary Table 2**). Furthermore, our study was limited to a small number of cases, so we analyzed patients with B-ALL and B-NHL together. In future analysis, if there are enough cases, patients with B-ALL and B-BHL should be analyzed separately, and more meaningful results may occur in this process.

## Data Availability

The original contributions presented in the study are included in the article/**Supplementary Material**. Further inquiries can be directed to the corresponding authors.
