# Supplementary material for: A viral infection prediction model for patients with r/r B-cell malignancies after CAR-T therapy: a retrospective analysis
Source: Front Oncol. 2025 Mar 21;15:1549809. doi: 10.3389/fonc.2025.1549809 (PMC11968754; doi:10.3389/fonc.2025.1549809)
Supplement: Supplementary file 1 [file Table1.docx]

Supplementary Table 1

|  | ALL | NHL | Total |
| --- | --- | --- | --- |
| CMV | 1 | 1 | 2 |
| EBV | 1 | 0 | 1 |
| HSV | 1 | 0 | 1 |
| HBV | 4 | 2 | 6 |
| Covid-19 and influenza | 2 | 3 | 5 |
| Muti-viral infection | 4 | 6 | 10 |
| Other viral infection | 1 | 0 | 1 |
| Total | 14 | 12 | 26 |

CMV: Cytomegalovirus; EBV: Epstein-Barr virus; HSV: herps simplex virus; HBV: Hepatitis B virus;

Supplementary Table 2

| ending | CRS | | | | | χ^2^ | p |
| --- | --- | --- | --- | --- | --- | --- | --- |
|  | 0 grade | 1 grade | 2 grade | 3 grade | Total | 1.350 | 0.717 |
| non-viral infection | 6 (30.0%) | 8 (40.0%) | 5 (25.0%) | 1 (5.0%) | 20(100.0%) |  |  |
| viral infection | 4 (16.0%) | 12 (48.0%) | 8 (32.0%) | 1 (4.0%) | 25 (100.0%) |  |  |
| Total | 10 (22.2%) | 20 (44.4%) | 13 (28.9%) | 2 (4.4%) | 45 (100.0%) |  |  |

P-values were determined using the Mann–Whitney U-test and χ2 test. The definitions of factors are same as Table1
